# Boston Letter

**Published:** 1879-08

**Authors:** 


ARTICLE XIV.

MESSRS. EDITORS:

Our State medical Society recently held an annual meeting, which was successful in all respects, and was made especially interesting by discussions on papers which were read before the general meeting. Papers on chosen subjects are read, by physicians who are appointed to this duty by a committee on medical essays. But in the previous years, at the annual meeting of 1878, for instance, the papers were listened to without a word of comment. This lack of responsiveness was criticised in the published report of the meeting. This year, however, President Lyman called upon various gentlemen for remarks, which gave rise to a spontaneous discussion. This feature is one which not only makes these meetings more valuable, but gratifies and partly compensates the readers, who, having expended much thought and research in the preparation of their papers, deserve recognition of this better sort.

Days before the meeting, the Fellows of the society are made acquainted with the subjects of papers to be read, and consequently there is no reason why they should not prepare themselves to speak. This increases the interest of papers which otherwise seem to fall flat, to the great disgust and discomfort of the gentlemen who have carefully and conscientiously written them.

The annual meeting of the Massachusetts Medical Society is held in some convenient hall, the executive officers having their quarters in the same building. That chosen for this year afforded abundant space, so that various surgical instrument makers and medical book publishers of this city and New York were able to make a fine exhibit of their wares; a most excellent thing for practitioners from the country, who of course but seldom have opportunity to examine the recent ingenuities in surgical and medical appliances.

At one o'clock on the second day of the meeting, the annual dinner is spread in our spacious music hall. The Fellows march in to the music of an orchestra, which is stationed in one of the galleries, and, with the exception of officers and invited guests, who are seated at special tables, they take their seats wherever they choose, at either of the long tables on the floor of the hall. An hour being given to the enjoyment of the dinner, which, this year, was particularly good, because nothing but cold dishes were provided, the anniversary chairman calls the meeting to order, and opens his budget of toasts, which are more or less *à propos*, according as his familiarity with the characteristics of respondents is extensive, and his own wit nimble and flexible.

The invited guests usually comprise the governor, the president of Harvard University, collector of the port, surgeon-general, a distinguised clergyman, who likewise officiates as annual chaplain, and representatives of the army and navy. The first gentleman called upon to respond, by courtesy, is the president of the society, then the president elect for the following year, next the governor, or in his absence his lieutenant. Toasts and responses are commonly interesting and provocative of mirth, for the speeches made at a doctor's dinner are apt to be amusing. The laymen present of course make use of what that body apparently considers its especial privilege, and so the antiquated changes upon the benefit to patients of the absence of their physicians, *et id genus omne*, are freely rung. At our dinner of this year about 650 gentlemen were present, of whom only five were laymen.

The affair lasts from one to four o'clock, fine music enlivens the occasion, and the companionable cigar of nearly every guest contributes to the cloud which slowly forms under the ceiling. After the dinner, and previous to the speeches, the Fellows form in groups and couples and distribute themselves about the galleries of the great hall. In every way the occasion is one of most agreeable enjoyment, especially to those faithful, hard working physicians from the country, who from year's end to year's end probably have no other relaxation from their wearying round of duties. It is hardly necessary to say that they, in common with the city Fellows, are proud of this grand old society, whose influence is so widely felt, and whose character deservedly stands so high.

The red letter feature of the annual meeting of the society is the annual gathering of its councilors, which body includes the officers and the various committees, and in whose care is placed the settlement of all the ethical, legal, financial and literary matters. For many years the councilors have met in contracted, inconvenient, stuffy rooms on temple place. This year they met, and will hereafter meet, in the beautiful hall of the Medical Library building, of which I gave you a description in a former letter. Henceforth, too, the archives and records of the society, heretofore kindly preserved by the Massachusetts Historical Society, will be deposited in this building, a safe having been provided for the purpose.

At this meeting discussions are as frank as they often are exciting. Last year the bone of contention was a new code of ethics, which even now has not reached a satisfactory completion. The woman question also came up, having been launched into the stream of discussion by a resolution from one of the district societies, which crystallized the topic by requesting the admission to the society of female practitioners "in good and regular standing." A committee appointed by the district society had canvassed the State, by addressing to Fellows of the parent society the question as to whether they advocated, or objected to the admission of women. The result, as embodied in their resolution, seemed to indicate a favorable response from the majority; but, since over 40 per cent. did not respond, the the councilors were not particularly credulous of the sanguine view taken by the committee in question. After some rather warm debate the matter was referred to a special committee, and has since been re-referred (this was at the winter meeting of the councilors), and finally at the recent gathering it was again postponed, because of the absence of Dr. H. W. Williams (one of the committee), who wished to express his views, but was detained by a most distressing accident to one of his children, which occurred only an hour before the time of meeting. The motion to postpone to the October meeting called forth some warm expressions, which implied that feeling and opinion, pro and con, were of a decided nature. This meeting gives large opportunity to the oratorically disposed, and since some of the councilors are politicians and members of the general court, their familiarity with parliamentary law is often made most amusingly evident. Now and then discussions become so earnest that the president's gavel is kept busy. Nevertheless, however warm the feeling of disputants may be, it is honest, generally serious, and its object is a sincere desire to promote the best welfare of the society.

We anticipate a certain degree of fervor in the discussion of the woman question at the October meeting. I shall take care to inform you of the result. It is a question which, at the present time, is interesting the laity as well as the medical faculty throughout the State.

You may have read that application for the admission of women to the Harvard medical school was made by interested parties, some fourteen months ago, and was the more calculated to awaken public interest from the fact that Miss Marion Hovey, as one of the trustees of a fund bequeathed by her father for benevolent purposes, proposed to present the sum of $10,000 to the school, on condition that "its advantages be offered to women on equal terms with men." The question of accepting the offer was referred to the overseers for their consideration and advice. They placed the communication in the hands of President Eliot and a committee, the chairman of which, Mr. Alexander Agassiz, issued a circular containing the following questions.

1. Are you in favor of admitting women to the medical school?

2. Are you in favor of admitting women on equal terms with men?

3. Are you in favor of a separate school for women?

4. If in favor of medical co-education, specify the subjects which, in your opinion, can be taught in common, and those in which men and women should receive separate instruction.

This circular was addressed to members of the Massachusetts Medical Society, a postal card being included, which bore numbered blanks for replies. What the exact result in negative and affirmative reply was, I do not know, but recently an adjourned meeting of the overseers of the university was holden, at which the matter was thoroughly discussed. Two reports, a majority and minority report, were presented. The former examined the question in all its bearings; and closed with a recommendation that the trust offered by Miss Hovey be accepted, under conditions which, briefly, were these:

"That, after the completion of a new building, women be admitted to the school, as an experiment, for a period of ten years.

"That they be not less than 22 years of age.

"That requisitions for admission, and the curriculum, be the same as for men.

"That the examination of men and women be identical.

"That nothing shall be countenanced which will lower the standard of the school, or affect the execution of the plans formed for its development.

"That the courses of lectures in which students take no active part be open to both sexes; that for personal instruction in laboratories and for recitation, men and women be separated; and that complete separation be made in such subjects as obstetrics, diseases of women and children, certain portions of anatomy, physiology and the like."

The sense of the minority report was, that while the admission of women to the profession of medicine is legitimate, perhaps called for, they will do far better in schools of their own, especially so since a plan requiring separation of the sexes in certain branches would be less advantageous to women than a school devoted exclusively to their sex.

It was finally voted "that the overseers find themselves unable to advise the President and Fellows to accept the generous proposal of Miss Hovey."

It was further voted, "that in the opinion of the overseers, it is expedient that, under suitable restrictions, women be instructed in medicine in Harvard University, in its medical school."

The latter was a qualifying vote which left some doubt as to the real feeling of the overseers. Nevertheless, the result was practically decisive against the admission of women to the school.

This is a cause of satisfaction to the faculty of the school, as well as to the majority of physicians here. Notwithstanding the sanguine tone of the petition, there is no popular demand for the coeducation of young men and women in medicine. Petitions of this nature are rarely trustworthy, for a mere signature does not always indicate the real sentiments of the signer, who may have given his name from sheer inability to refuse, and probably without due and careful forethought.

I merely present the case as it stands and without further comment, preferring to await the doings and opinions of the councilors' meeting of the State society. It will probably do much to settle the whole matter, so far as Massachusetts is concerned.

Before I close, I cannot deny myself the pleasure of giving you the remarkable obituary notice of the death of Drs. Jacob Bigelow and J. B. S. Jackson, which was read at the late meeting of councilors, by Dr. Calvin Ellis, the chairman of a committee appointed for the purpose:

"When at this, our annual meeting, we call the roll and receive no response from

JACOB BIGELOW, or
JOHN BARNARD SWETT JACKSON,

"whose lives were full of noble labor, we are reminded that the mere mention of their names is a fuller expression of a sense of loss than any formal phrase of ours."

The unusual force and beauty of this testimonial created a deep mpression. * *

BOSTON, July 12, 1879.

